# Activation of β3-adrenoceptor increases the number of readily releasable glutamatergic vesicle via activating Ca^2+^/calmodulin/MLCK/myosin II pathway in the prefrontal cortex of juvenile rats

**DOI:** 10.1038/s41598-021-97769-4

**Published:** 2021-09-15

**Authors:** Xing Wang, Xuan Sun, Hou-Cheng Zhou, Fei Luo

**Affiliations:** 1 School of Life Science, Nanchang University, Nanchang, 330031 China; 2 Institute of Neurobiology and State Key Laboratory of Medical Neurobiology, Institutes of Brain Science, Fudan University, Shanghai, 200032 China; 3 Center for Neuropsychiatric Diseases, Institute of Life Science, Nanchang University, Nanchang, 330031 China

**Keywords:** Synaptic transmission, Vesicle trafficking

## Abstract

It is well known that β3-adrenoceptor (β3-AR) in many brain structures including prefrontal cortex (PFC) is involved in stress-related behavioral changes. SR58611A, a brain-penetrant β3-AR subtypes agonist, is revealed to exhibit anxiolytic- and antidepressant-like effects. Whereas activation of β3-AR exerts beneficial effects on cognitive function, the underlying cellular and molecular mechanisms have not been fully determined. In this study, whole cell patch-clamp recordings were employed to investigate the glutamatergic transmission of layer V/VI pyramidal cells in slices of the rat PFC. Our result demonstrated that SR58611A increased AMPA receptor-mediated excitatory postsynaptic currents (AMPAR-EPSCs) through activating pre-synaptic β3-AR. SR58611A enhanced the miniature EPSCs (mEPSCs) and reduced paired-pulse ratio (PPR) of AMPAR-EPSCs suggesting that SR58611A augments pre-synaptic glutamate release. SR58611A increased the number of readily releasable vesicle (*N*) and release probability (*Pr*) with no effects on the rate of recovery from vesicle depletion. Influx of Ca^2+^ through L-type Ca^2+^ channel contributed to SR58611A-mediated enhancement of glutamatergic transmission. We also found that calmodulin, myosin light chain kinase (MLCK) and myosin II were involved in SR58611A-mediated augmentation of glutamate release. Our current data suggest that SR58611A enhances glutamate release by the Ca^2+^/calmodulin/MLCK/myosin II pathway.

## Introduction

Previous studies from human and animals have revealed a relationship between norepinephrine (NE) system function and stress-induced cognition^1, 2^, many lines of evidence have indicated that β-adrenoceptor (β-AR) subtype is involved in stress-related behavioral outcomes. For example, high level of NE release during stress impairs prefrontal cortex (PFC) functions via β1-AR^3^. In contrast, injection of β2-AR in monkeys and rats exerts beneficial effects on working memory^4^. SR58611A, a selective brain-penetrant β3-AR agonist, is used as a novel treatment strategy for stress-related disorders such as anxiety and depression^5, 6^.

Experiments utilizing reverse transcription/PCR approach have demonstrated the existence of β3-AR mRNA in independent regions including amygdala, hippocampus as well as cerebral cortex in both human and rats^7–9^. The neurochemical action of SR58611A is well characterized. For example, administration of SR58611A enhances release of NE and 5-HT from pre-synaptic nerve terminal, resulting in elevated concentration of NE and 5-HT at synapse cleft in several brain regions in rats, but has no effect on dopaminergic neurotransmission^10^. However, the potential neural mechanisms underlying modulation of glutamatergic system induced by SR58611A are elusive yet.

Prefrontal cortex (PFC) is a critical brain structure mediating various stress-induced disorders such as attention deficit hyperactivity disorder (ADHD), post-traumatic stress disorder (PTHD), and schizophrenia^11–13^. Glutamatergic transmission in PFC deep (layer V/VI) pyramidal neurons exerts an important role in controlling PFC activity and cognitive function^14, 15^, and disturbed glutamate receptor has been shown to mediate many mental disorders^16^. The present study investigates the role of SR58611A in glutamatergic transmission and its potential cellular and molecular mechanism in the PFC. Our results suggest that activation of pre-synaptic β3-AR results in Ca^2+^ influx via L-type Ca^2+^ channel. Ca^2+^ then activates calmodulin leading to the activation of MLCK. Phosphorylation of myosin light chain changes the conformation of myosin II facilitating the delivery of synaptic vesicles from the reserve pool to the readily releasable pool to increase glutamate release.

## Results

### SR58611A increases AMPA receptor-mediated EPSCs via activation of β3-AR

**Figure 1:**
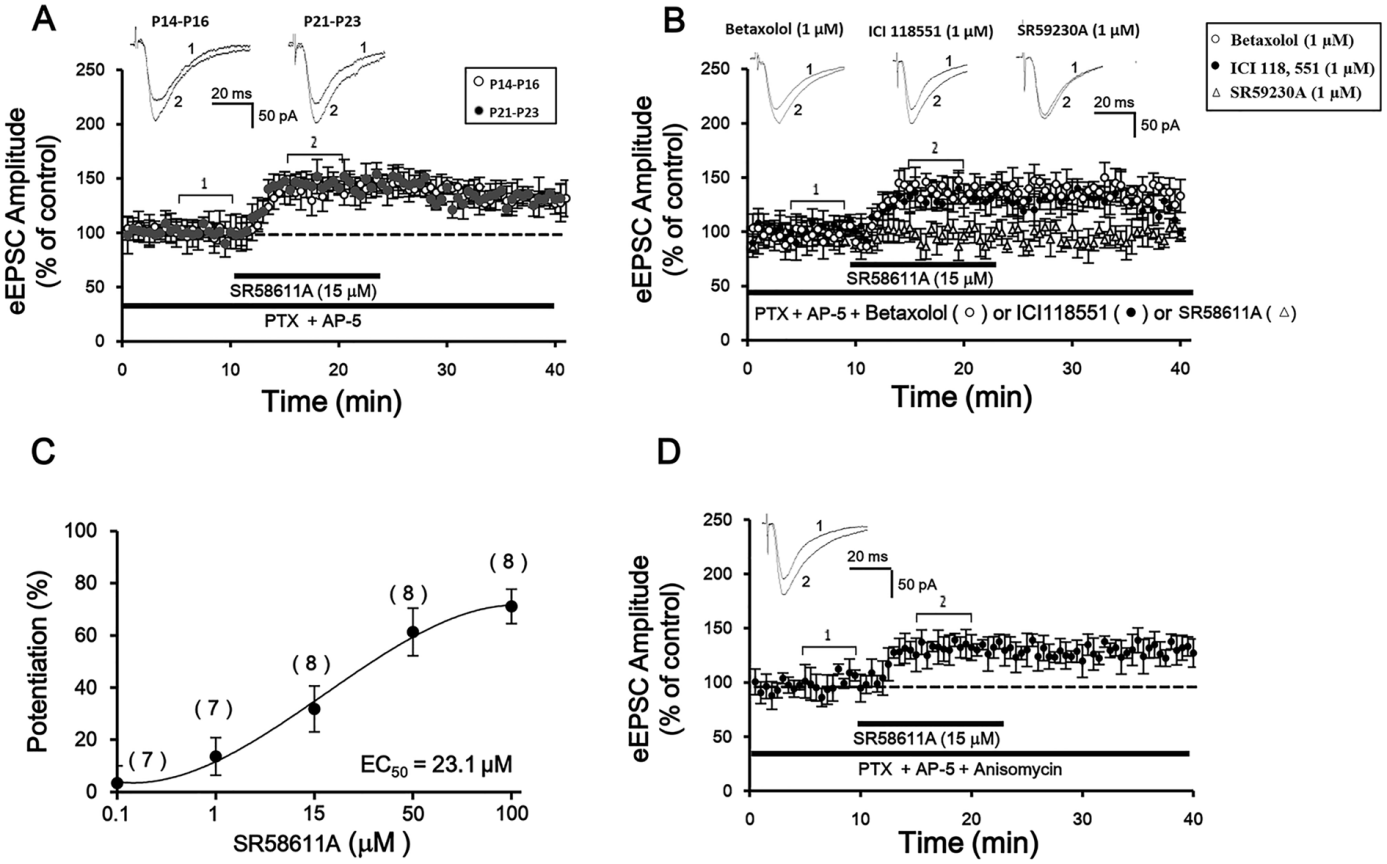
Stimulation of β3-AR facilitates glutamatergic transmission. (**A**) Summary of experiments showed that SR58611A (15 µM) enhances AMPAR-EPSCs in the presence of GABA_A_ receptor antagonist PTX (100 µM) and NMDA receptor antagonist AP-5 (50 µM) at a holding potential of − 70 mV. Sample traces were averages of ten consecutive AMPAR-EPSCs recorded at the time courses indicated by the numbers on the graph. Horizontal bars denoted the period of delivery of SR58611A. (**B**) Summary of experiments showed that SR58611A-induced potentiates of AMPAR-EPSCs were blocked by β3-AR antagonist SR59230A (1 µM), but not by β1-AR antagonist betaxolol (1 µM) or β2-AR antagonist ICI118551 (1 µM). (**C**) A SR58611A concentration–response curve. Number of neurons used was showed at the top of each group. (**D**) Summary of experiments showed that application of the transcriptional inhibitor, anisomycin (10 µM) failed to block SR58611A-induced increases in AMPAR-EPSCs.

We first examined the effects of SR58611A (15 µM) on glutamatergic transmission onto layer V/VI pyramidal neurons of the PFC via recording AMPAR-EPSCs in the presence of PTX (100 µM) and AP-5 (50 µM) at a holding potential of − 70 mV. Layer V/VI pyramidal cells had a prominent apical dendrite, and displayed a spike frequency adaptability property when depolarization currents were injected as we described previously^17^. As illustrated in Fig. 1A, the amplitude of AMPAR-EPSCs began to increase gradually and reached maximal effect in approximately 5 min after the beginning of SR58611A (15 µM) application (Fig. 1A, potentiation of 32.3 ± 6.3%, n = 8 cells from six rats, P < 0.05 vs. control, paired t-test). Because above experiments were conducted in slices from P14-P16 rats, we next examined the effect of SR58611A (15 µM) on AMPAR-EPSCs by using P21-P23 rats. As shown in Fig. 1A, SR58611A (15 µM) still increased AMPAR-EPSCs at P21-P23 (Fig. 1A, potentiation of 30.8 ± 5.9%, n = 10 cells from seven rats, P < 0.05 vs. control, paired t-test). One-way analysis of variance (ANOVA) did not revealed a significant main effect of rat age on SR58611A-induced enhancement of AMPAR-EPSCs (Fig. 1A, F_1,17_ = 0.63, P > 0.05). Subsequent post-hoc comparison results also showed that the extent of potentiation effect induced by SR58611A (15 µM) was not significantly different between P14-P16 group and P21-P23 group (P > 0.05). Because SR58611A (15 µM) exerted equal effects on enhancement of AMPAR-EPSCs at P14-16 and P21-23, we used P14-23 rats for the remaining studies unless otherwise stated.

Because SR58611A displays low efficacy and potency at the rat and human β1- and β2-AR, it is possible that SR58611A-induced facilitation effect is mediated via β1- and/or β2-AR. We then examined the effect of SR58611A on AMPAR-EPSCs in the extracellular of presence of β1-AR antagonist betaxolol (1 µM), β2-AR antagonist ICI118551 (1 µM) or β3-AR antagonist SR59230A (1 µM), respectively. Under these situations, betaxolol (1 µM) or ICI118551 (1 µM) did not abolish SR58611A-enhanced AMPAR-EPSCs (Fig. 1B, betaxolol: potentiation of 30.6 ± 6.7%, n = 8 cells from seven rats, P < 0.05 vs. control, paired t-test, ICI118551: potentiation of 29.4 ± 6.2%, n = 8 cells from six rats, P < 0.05 vs. control, paired t-test), but SR59230A (1 µM) almost completely blocked SR58611A-enhanced AMPAR-EPSCs (Fig. 1B, potentiation of 4.7 ± 4.1%, n = 7 cells from six rats, P > 0.05 vs. control, paired t-test). One-way ANOVA revealed a significant main effect of antagonist treatment on SR58611A-induced enhancement of AMPAR-EPSCs (F_3, 30_ = 23.6, P < 0.01). Subsequent post-hoc comparison results showed that the extent of potentiation effect induced by SR58611A (15 µM) in the presence of betaxolol (1 µM) or ICI118551 (1 µM) was not significantly different with that induced by SR58611A (15 µM) alone (P > 0.05), but SR58611A-induced enhancement of AMPAR-EPSCs in the presence of SR59230A (1 µM) was significantly weaker than that induced by SR58611A (15 µM) alone (P < 0.01). These results suggest that SR58611A-enhanced glutamatergic transmission is mediated via β3-AR rather than β1- and β2-AR. The extent of potentiation effect of SR58611A (15 µM) on AMPAR-EPSCs was dose-dependent, with the EC_50_ being 23.1 µM in our recording condition (Fig. 1C).

Because the maximal effect of SR58611A (15 µM) was observed approximately 5 min after the beginning of SR58611A (15 µM) application, we next probed whether protein synthesis participates in SR58611A-mediated enhancement of AMPAR-EPSCs through adding a transcription inhibitor anisomycin (10 µM) in the bath solution. Under this condition, SR58611A (15 µM) still increased AMPAR-EPSCs (Fig. 1D, potentiation of 29.2 ± 6.4%, n = 8 cells from seven rats, P < 0.05 vs. control, paired t-test), the extent of potentiation effect was not significantly different with that induced by SR58611A (15 µM) alone (F_1, 15_ = 0.87, P > 0.05), suggesting that protein synthesis is not necessary for SR58611A-enhanced glutamatergic transmission.

### SR58611A facilitates pre-synaptic glutamate release

**Figure 2:**
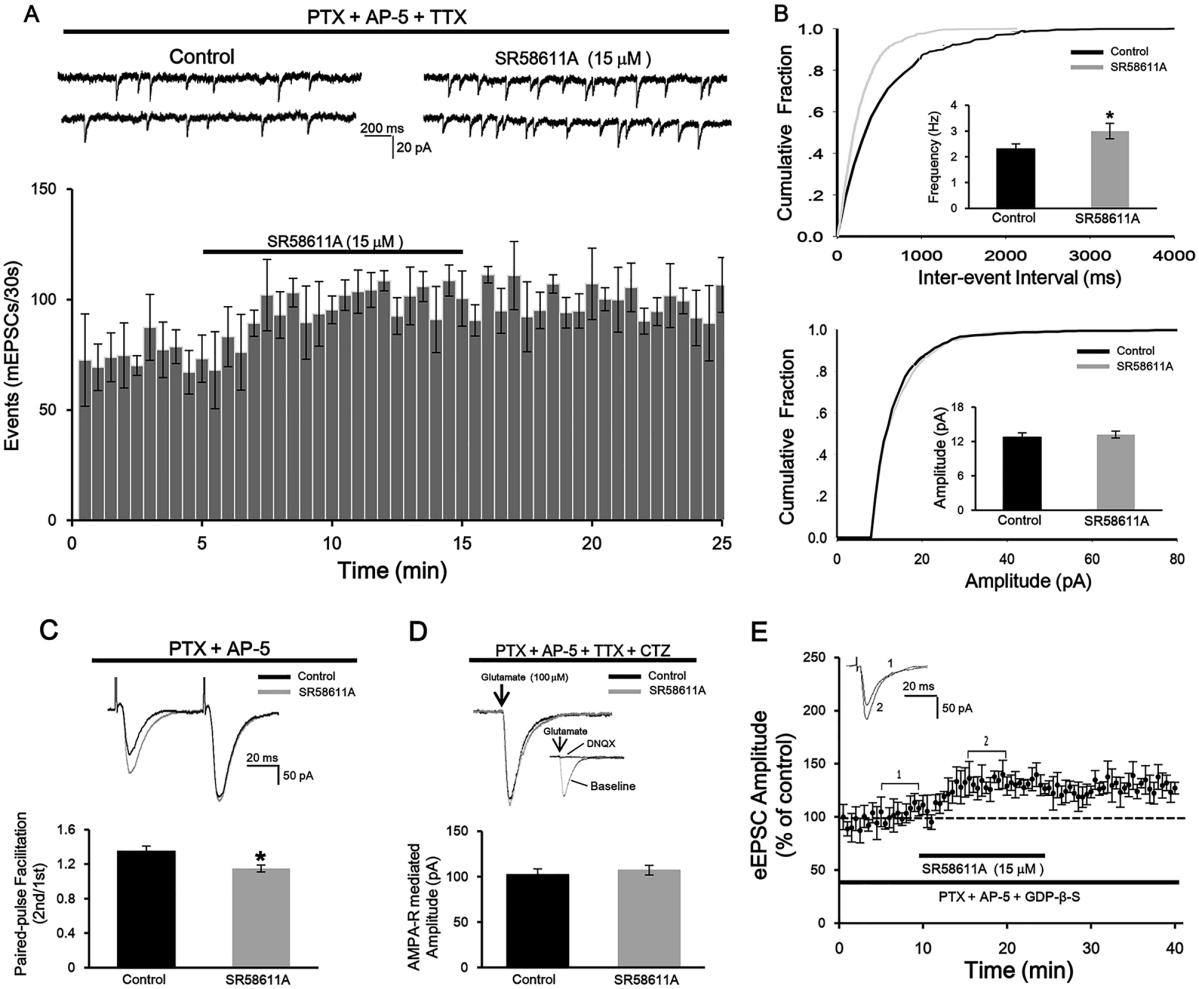
Stimulation of β3-AR facilitates AMPAR-EPSCs via increasing pre-synaptic glutamate release. (**A**) Upper: A typical trace of mEPSCs recorded before (left) and during (right) the application of SR58611A (15 µM) in the presence of PTX (100 µM), AP-5 (50 µM) and TTX (1 µM) at a holding potential of − 70 mV. Lower: Time course of the SR58611A-induced increase in mEPSC frequency averaged from 7 cells. (**B**) Upper: Cumulative distribution of inter-event intervals and histograms showed that SR58611A (15 µM) reduced mEPSC frequency. Lower: Cumulative distribution of amplitude and histograms showed that the SR58611A (15 µM) had no effect on mEPSC amplitude. (**C**) SR58611A (15 µM) decreased the paired-pulse ratio (PPR = EPSC2/EPSC1, EPSC1and EPSC2 were the EPSCs evoked by two stimuli at an interval of 50 ms) of AMPAR-EPSCs. (**D**) SR58611A (15 µM) did not changed post-synaptic AMPA receptor-mediated currents evoked by puff application of glutamate (100 µM) in the extracellular presence of PTX (100 µM), AP-5 (50 µM), TTX (1 µM) and CTZ (10 µM). Inset indicated that bath application of AMPA receptor antagonist DNQX (25 µM) completely blocked glutamate-induced currents. *P < 0.05 vs. control. (**E**) Summary of experiments showed that application of SR58611A (15 µM) still significantly increased AMPAR-EPSCs amplitudes in the intracellular presence of a general G-protein inactivator GDP-β-S (1 mM).

Given that SR58611A-induced enhancement of glutamatergic transmission could come from an increase in pre-synaptic glutamate release and/or an up-regulation of post-synaptic AMPA receptor function. We next differentiated between the pre-synaptic and post-synaptic effects of SR58611A. First, we examined the effect of SR58611A (15 µM) on mEPSCs because an alteration in pre-synaptic glutamate release usually accompanied by a change of mEPSC frequency. As shown in Fig. 2A, bath application of SR58611A (15 µM) significantly increased mEPSC frequency (Fig. 2A and B, potentiation of 26.1 ± 4.7%, n = 7 cells from six rats, P < 0.05 vs. control, paired t-test) rather than amplitude (Fig. 2A and B, potentiation of 3.4 ± 2.4%, n = 7 cells from six rats, P > 0.05 vs. control, paired t-test). Second, we examined the paired-pulse ratio (PPR) induced by SR58611A (15 µM) because the change of PPR reflects an alteration in pre-synaptic glutamate release. As illustrated in Fig. 2C, SR58611A (15 µM) significantly reduced PPR (Fig. 2C, reduction of 21.1 ± 4.4%, n = 7 cells from six rats, P < 0.05 vs. control, paired t-test). Third, we recorded glutamate-induced currents through puff application of exogenous glutamate (100 µM) onto the cell body of the recorded neuron in the extracellular presence of PTX (100 µM), AP-5 (50 µM), TTX (1 µM) and CTZ (10 µM). As illustrated in Fig. 2D inset, AMPA receptor antagonist DNQX (25 µM) completely counteracted glutamate-induced currents, indicating that the currents were mediated via AMPA receptor. Application of SR58611A (15 µM) had no effect on glutamate-induced currents (Fig. 2D, potentiation of 2.4 ± 1.9%, n = 7 cells from six rats, P > 0.05 vs. control, paired t-test), suggesting that SR58611A (15 µM) did not directly regulate post-synaptic AMPA receptor function. Taken together, these results indicate that SR58611A (15 µM) increases glutamatergic transmission via facilitating pre-synaptic glutamate release rather than directly modulating post-synaptic AMPA receptor function.

We then investigated whether SR58611A (15 µM) increases glutamate release via activating post-synaptic β3-AR because SR58611A (15 µM) may act on post-synaptic β3-AR to generate retrograde messengers to increase glutamate release. To test this possibility, a general G-protein inactivator GDP-β-S (1 mM) was contained in the recording pipettes to block post-synaptic G-protein activity, and then blocked post-synaptic β3-AR function. Under this condition, we found that SR58611A-induced potentiation of AMPAR-EPSCs was still observed (Fig. 2E, potentiation of 27.7 ± 6.2%, n = 8 cells from seven rats, P < 0.05 vs. control, paired t-test), the extent of potentiation effect was not significantly different with that induced by SR58611A (15 µM) alone (F_1,15_ = 0.78, P > 0.05), excluding the involvement of post-synaptic β3-AR. These results suggest that SR58611A (15 µM) increases glutamate release via activating pre-synaptic β3-AR.

### SR58611A-induced enhancement of glutamate release requires elevation of intracellular Ca^2+^ concentration

**Figure 3:**
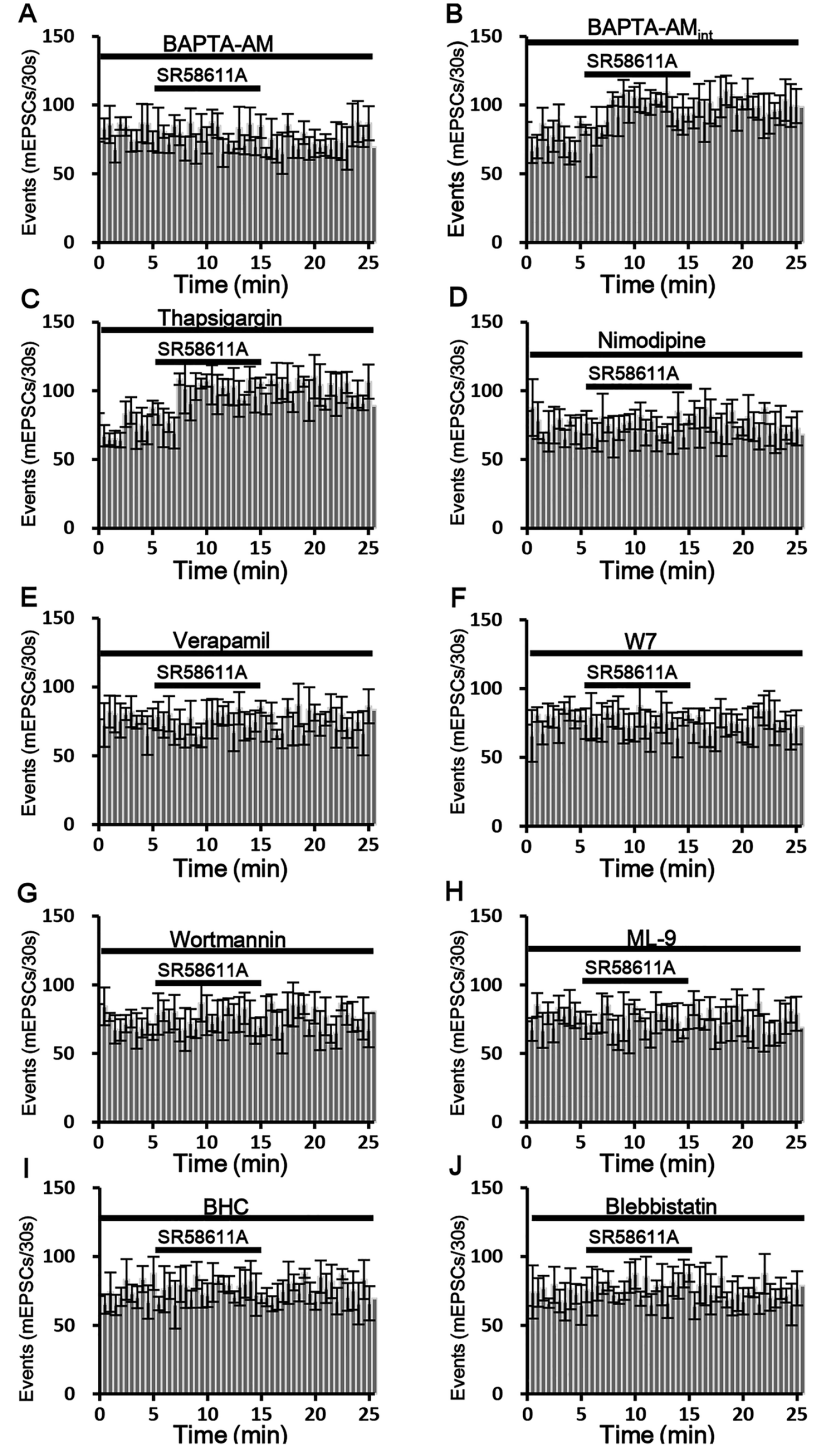
SR58611A-enhanced glutamate release requires the involvement of the Ca^2+^ /calmodulin/MLCK/myosin II pathway. (**A**) Bath application of BAPTA-AM (100 µM), a selective chelator of Ca^2+^, blocked SR58611A-induced augmentation of mEPSC frequency. (**B**) Intracellular perfusion of BAPTA-AM (100 µM) via the recording pipettes did not influence SR58611A-mediated augmentation of mEPSC frequency. (**C**) Bath application of the sarcoplasmic reticulum Ca^2+^-ATPase inhibitor thapsigargin (10 µM) did not abolish SR58611A-induced enhancement of mEPSC frequency. (**D**) Bath application of the L-type Ca^2+^ channel inhibitor nimodipine (10 µM) abolished SR58611A-enhanced mEPSC frequency. (**E**) Bath application of another L-type Ca^2+^ channel blocker verapamil (10 µM) counteracted SR58611A-induced increases in mEPSC frequency. (**F**) Bath application of the calmodulin inhibitor W7 (50 µM) blocked SR58611A-induced increases in mEPSC frequency. (**G**) Bath application of the MLCK inhibitor wortmannin (10 µM) blocked SR58611A-induced increases in mEPSC frequency. (**H**) Bath application of the ML-9 (10 µM), another MLCK inhibitor, abolished SR58611A-induced increases in mEPSC frequency. (**I**) Bath application of a general myosin inhibitor BHC (1 µM) blocked SR58611A-induced increases in mEPSC frequency. (**J**) Bath application of a specific myosin II inhibitor blebbistatin (1 µM) abolished SR58611A-induced increases in mEPSC frequency.

Because elevation of intracellular Ca^2+^ can result in increases in glutamate release^18^, we next investigated whether the elevation of intracellular Ca^2+^ concentration is involved in SR58611A-enhanced glutamate. As shown in Fig. 3A, bath application of the Ca^2+^ chelator BAPTA-AM (100 µM) significantly blocked SR58611A-enhanced mEPSC frequency (Fig. 3A, reduction of 2.5 ± 1.9%, n = 7 cells from six rats, P > 0.05 vs. baseline in the presence of BAPTA-AM alone, paired t-test). One-way ANOVA and subsequent post-hoc comparison results showed that SR58611A-induced extent of potentiation effect in the presence of BAPTA-AM (100 µM) was significantly weaker than that induced by SR58611A (15 µM) alone (F_1,14_ = 23.0, P < 0.01), suggesting that SR58611A-mediated enhancement of AMPAR-EPSCs is dependent on an increase in intracellular Ca^2+^. We further tested whether the effect of BAPTA-AM comes from pre-synaptic and/or post-synaptic sites, BAPTA-AM (100 µM) was included in the recording pipettes to act on only post-synaptic site. Under this circumstance, SR58611A-enhanced mEPSC frequency was still observed (Fig. 3B, potentiation of 30.4 ± 5.7%, n = 7 cells from six rats, P < 0.05 vs. baseline in the presence of BAPTA-AM alone, paired t-test). One-way ANOVA and subsequent post-hoc comparison results showed that SR58611A-induced extent of potentiation effect in the presence of BAPTA-AM (100 µM) was not significantly different with that induced by SR58611A (15 µM) alone (F_1,14_ = 0.66, P > 0.05). Taken together, these results demonstrate that elevation of pre-synaptic, not post-synaptic, Ca^2+^ is responsible for SR58611A-induced increases in glutamate release.

Given that increases in the intracellular Ca^2+^ concentration could arise from release from intracellular Ca^2+^ stores and/or extracellular Ca^2+^ influx, we next tested whether intracellular Ca^2+^ release from Ca^2+^ stores is involved in SR58611A-enhanced glutamate release. We pre-treated slice 30 min with a sarcoplasmic reticulum Ca^2+^-ATPase inhibitor thapsigargin (10 µM), and then recorded SR58611A-induced effect on mEPSCs in the extracellular presence of the same concentration of thapsigargin. Under this circumstance, SR58611A (15 µM) still significantly increased mEPSC frequency (Fig. 3C, potentiation of 31.5 ± 4.2%, n = 8 cells from six rats, P < 0.05 vs. baseline in the presence of thapsigargin alone, paired t-test). One-way ANOVA and subsequent post-hoc comparison results showed that SR58611A-induced extent of potentiation effect in the presence of thapsigargin (10 µM) was not significantly different with that induced by SR58611A (15 µM) alone (F_1,15_ = 0.65, P > 0.05), excluding the involvement of intracellular Ca^2+^ stores. These results suggest that elevation of Ca^2+^ in the pre-synaptic terminals in response to SR58611A (15 µM) must be from the extracellular side.

### L-type Ca^2+^ channel contributes to SR58611A-mediated enhancement of glutamate release

Because β3-AR has been reported to interact with L-type Ca^2+^ channel^19^, we therefore examined whether L-type Ca^2+^ channel participates in SR58611A-induced increase in glutamate release. Slices were pre-incubated with nimodipine (10 µM), a specific L-type Ca^2+^ channel blocker, and the same concentration of nimodipine was included in the bath solution. Under this condition, SR58611A (15 µM) did not significantly increase mEPSC frequency (Fig. 3D, potentiation of 6.3 ± 4.2%, n = 7 cells from five rats, P > 0.05 vs. baseline in the presence of nimodipine alone, paired t-test). One-way ANOVA and subsequent post-hoc comparison results showed that SR58611A-induced extent of potentiation effect in the presence of nimodipine (10 µM) was significantly weaker than that induced by SR58611A (15 µM) alone (F_1,14_ = 14.3, P < 0.01). Likewise, another L-type Ca^2+^ channel blocker verapamil (10 µM) also blocked SR58611A-enhanced mEPSC frequency (Fig. 3E, potentiation of 5.0 ± 3.8%, n = 7 cells from six rats, P > 0.05 vs. baseline in the presence of verapamil alone, paired t-test). One-way ANOVA and subsequent post-hoc comparison results showed that SR58611A-induced extent of potentiation effect in the presence of verapamil (10 µM) was significantly weaker than that induced by SR58611A (15 µM) alone (F_1,14_ = 15.5, P < 0.01). These data suggest that Ca^2+^ influx via L-type Ca^2+^ channel contributes to SR58611A-induced enhancement of glutamate release.

Given that calmodulin is a potential downstream target of intracellular Ca^2+^, we next studied the effect of calmodulin on SR58611A-enhanced glutamate release. As illustrated in Fig. 3F, bath application of SR58611A (15 µM) failed to enhance mEPSC frequency when the calmodulin inhibitor W7 (50 µM) was included in the extracellular solution (Fig. 3F, potentiation of 5.7 ± 3.1%, n = 7 cells from six rats, P > 0.05 vs. baseline in the presence of W7 alone, paired t-test). One-way ANOVA and subsequent post-hoc comparison results showed that SR58611A-induced extent of potentiation effect in the presence of W7 (50 µM) was significantly weaker than that induced by SR58611A (15 µM) alone (F_1,14_ = 14.7, P < 0.01), suggesting that calmodulin is necessary for SR58611A-enhanced glutamate release.

It is well known that calmodulin-dependent MLCK plays an important role in the process of glutamate release^20^, we next investigated the roles of MLCK in SR58611A-induced increase in glutamate release. As illustrated in Fig. 3G, SR58611A-induced potentiation of mEPSC frequency was not observed in the extracellular presence of MLCK inhibitor wortmannin (10 µM) (Fig. 3G, potentiation of 4.2 ± 2.6%, n = 7 cells from six rats, P > 0.05 vs. baseline in the presence of wortmannin (10 µM) alone, paired t-test). One-way ANOVA and subsequent post-hoc comparison results showed that SR58611A-induced extent of potentiation effect in the presence of wortmannin (10 µM) was significantly weaker than that induced by SR58611A (15 µM) alone (F_1,14_ = 17.1, P < 0.01). Given that wortmannin (10 µM) lacks the specificity for MLCK^21^, we used another MLCK inhibitor ML-9 to examine the role of MLCK in SR58611A-induced increase in glutamate release. As shown in Fig. 3H, SR58611A (15 µM) did not significantly enhance mEPSC frequency in the presence of ML-9 (10 µM) (Fig. 3H, potentiation of 2.9 ± 2.2%, n = 7 cells from six rats, P > 0.05 vs. baseline in the presence of ML-9 (10 µM) alone, paired t-test). One-way ANOVA and subsequent post-hoc comparison results showed that SR58611A-induced extent of potentiation effect in the presence of ML-9 (10 µM) was significantly weaker than that induced by SR58611A (15 µM) alone (F_1,14_ = 18.6, P < 0.01), further indicating that MLCK is involved in SR58611A-induced enhancement of glutamate release.

Because MLCK has been reported to be implicated in regulating the function of myosin^20^, we next investigated whether SR58611A-enhanced glutamate release also requires myosin function. Slice was pre-incubated with a general myosin inhibitor BHC (1 µM), and the same concentration of BHC was contained in the bath solution. Under this condition, SR58611A (15 µM) significantly blocked mEPSC frequency (Fig. 3I, potentiation of 5.3 ± 3.3%, n = 7 cells from six rats, P > 0.05 vs. baseline in the presence of BHC (1 µM) alone, paired t-test). One-way ANOVA and subsequent post-hoc comparison results showed that SR58611A-induced extent of potentiation effect in the presence of BHC (1 µM) was significantly weaker than that induced by SR58611A (15 µM) alone (F_1,14_ = 15.2, P < 0.01). Whereas myosin contains various isoforms including the class I and the class II subtype, the class II families have been implicated in the modulation of synaptic transmission^22^. We then tested whether myosin II participates in SR58611A-enhanced glutamate release. Slice was pre-incubated with a specific myosin II inhibitor blebbistatin (1 µM), and the same concentration of blebbistatin was included in the bath solution. Under these circumstances, SR58611A-enhanced mEPSC frequence was not observed (Fig. 3J, potentiation of 4.1 ± 3.1%, n = 7 cells from six rats, P > 0.05 vs. baseline in the presence of blebbistatin (1 µM) alone, paired t-test). One-way ANOVA and subsequent post-hoc comparison results showed that SR58611A-induced extent of potentiation effect in the presence of blebbistatin (1 µM) was significantly weaker than that induced by SR58611A (15 µM) alone (F_1,14_ = 19.0, P < 0.01). Taken together, these results indicate that SR58611A-induced increase in glutamate release requires calmodulin, MLCK and myosin II function, probably via the Ca^2+^/calmodulin/MLCK/myosin II pathway.

### SR58611A increases the number of releasable glutamatergic vesicles and release probability via activating the Ca^2+^/calmodulin/MLCK/myosin II pathway

**Figure 4:**
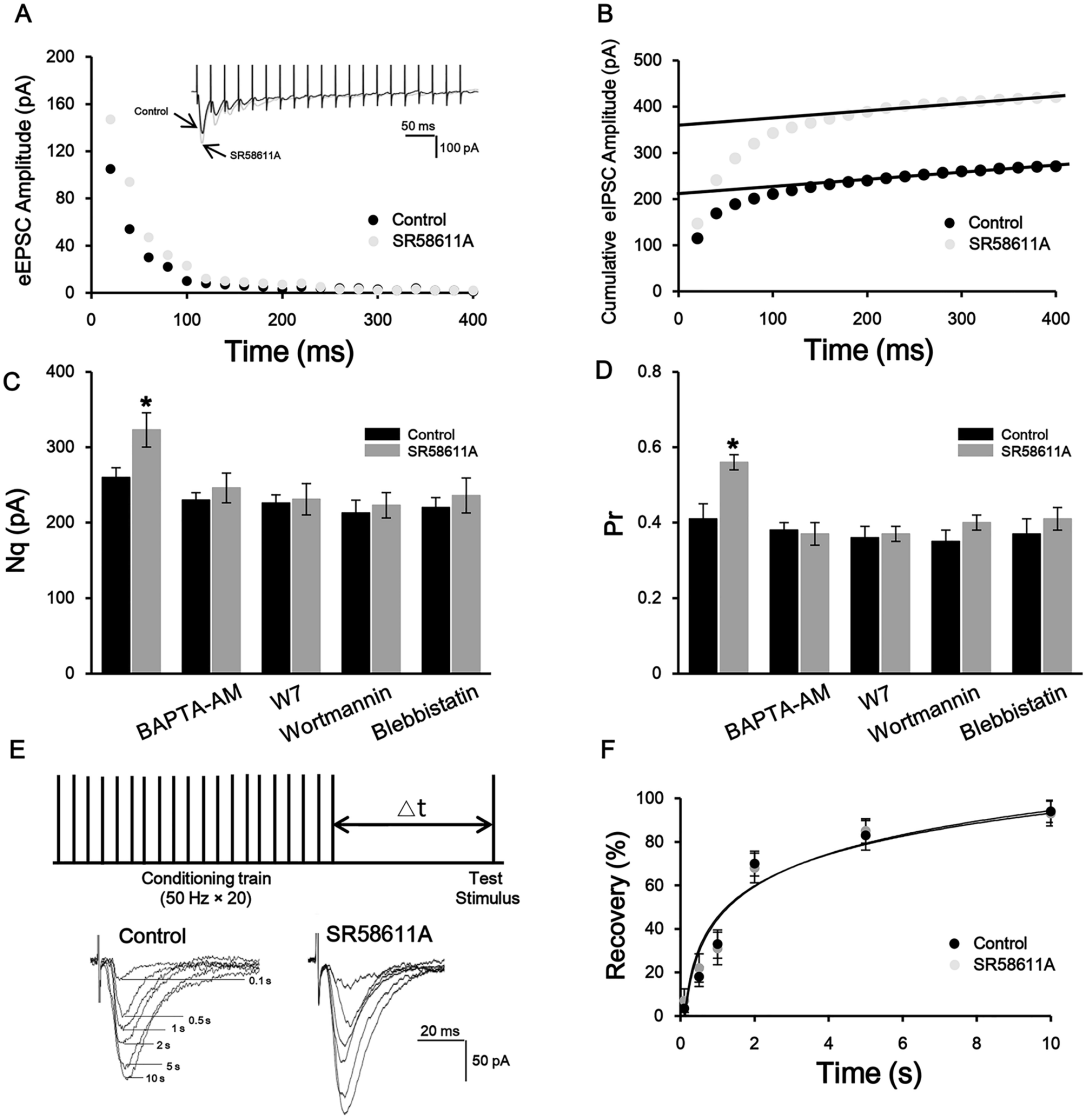
SR58611A increases glutamate release probability and the number of readily releasable vesicles via activating the Ca^2+^/calmodulin/MLCK/myosin II pathway. (**A**) Depressions of EPSCs during trains (20 stimuli) of 50 Hz stimulation before and during application of SR58611A (15 µM). The amplitude of EPSCs by each stimulus was measured by resetting the base line each time at a point within 0.5 ms before the beginning of each stimulation artifact. (**B**) Cumulative amplitudes of EPSCs during the 50 Hz train before and during SR58611A (15 µM) application. Amplitudes of EPSCs from 16 to 20th were fitted with a linear regression line and extrapolated to time 0 for estimating the readily releasable pool size. (**C**) Application of SR58611A (15 µM) increased mean number of releasable vesicles (*N*) multiplied by mean quantal size (*q*), and SR58611A-enhanced *Nq* was abolished in the presence of BAPTA-AM (100 µM), W7 (50 µM), wortmannin (10 µM), or blebbistatin (1 µM), respectively. (**D**) Application of SR58611A (15 µM) increased mean release probability (*P*_*r*_), and SR58611A-enhanced Pr was not observed in the presence of BAPTA-AM (100 µM), W7 (50 µM), wortmannin (10 µM), or blebbistatin (1 µM), respectively. (**E**) Upper: experimental protocol. A conditioning train (20 stimuli at 50 Hz) was followed by a test stimulus. The intervals between the end of the conditioning train and the beginning of the test stimulus were 0.1 s, 0.5 s, 1 s, 2 s, 5 s or 10 s. The interval between each sweep was 30 s to allow the refilling of the vesicles. Lower: EPSCs evoked by the test pulse from the same synapse at different intervals were aligned and superimposed before (left) and during (right) application of SR58611A (15 µM). (**F**) Time course of recovery from depletion before and during the application of SR58611A (15 µM) expressed as percentage recovery = (I_test_ − I_s_)/(I_1st_ − I_s_) × 100, where I_test_ is the EPSCs evoked by the test pulse, I_s_ is the steady-state current left after the conditioning train (the average of the last 5 EPSCs evoked by the conditioning train), I_1st_ is the EPSCs evoked by the 1st stimulus of the conditioning train. Data before and during the application of SR58611A (15 µM) from 7 cells were fitted by a single exponential function. *P < 0.05 versus control.

Whereas our above results have revealed that SR58611A (15 µM) increases glutamate release, several pre-synaptic mechanisms could lead to this facilitation effect such as an increase in the size of the readily releasable pool (*Nq*, the product of the number of readily releasable quanta, *N*, and the quantal size, *q*) and/or release probability (*P*_*r*_)^23, 24^. We next assessed SR58611A-induced changes in *Nq* and *P*_*r*_ utilizing a high-frequency stimulation (20 stimuli at 50 Hz) protocol. EPSC underwent a significant depression and reached a steady low level during this high-frequency stimulation. Figure 4A showed the EPSC trains before and during the SR58611A (15 µM) application (Fig. 4A). *N* multiplied by mean quantal size (*q*) could be estimated from zero time intercept of a line fitted to a cumulative amplitude plot of EPSC, and *P*_*r*_ can be estimated from the first EPSC amplitude divided by *Nq* (Fig. 4B). We found that SR58611A (15 µM) increased *Nq* (Fig. 4C, potentiation of 22.7 ± 4.9%, n = 7 cells from six rats, P < 0.05 vs. control, paired t-test) as well as *P*_*r*_ (Fig. 4D, potentiation of 27.2 ± 4.1%, n = 7 cells from seven rats, P < 0.05 vs. control, paired t-test). Because mEPSC amplitude represents quantal size (*q*)^25^, and our above finding demonstrates that SR58611A (15 µM) does not influence the quantal size (Fig. 2B). Taken together, these results indicate that SR58611A (15 µM) increases the number of readily releasable quanta (*N*) and release probability (*P*_*r*_).

To test whether the Ca^2+^/calmodulin/MLCK/myosin II pathway is involved in the SR58611A-induced facilitation effect of *N* and *P*_*r*_. BAPTA-AM (100 µM), W7 (50 µM), wortmannin (10 µM), or blebbistatin (1 µM) was included in the bath solution respectively, under these circumstance, SR58611A-enhanced *N* and *P*_*r*_ was not observed in the presence of BAPTA-AM (100 µM), W7 (50 µM), wortmannin (10 µM), or blebbistatin (1 µM), respectively (Fig. 4C,D), suggesting that the Ca^2+^/calmodulin/MLCK/myosin II pathway participates in SR58611A-induced enhancement of the number of readily releasable quanta (*N*) and release probability (*P*_*r*_).

Increases in the size of the readily releasable pool can occur with or without a concomitant increase in the rate of vesicle replenishment, we next studied the effect of SR58611A (15 µM) on recovery rate from vesicle depletion according to a protocol described previously^26^, a train of high-frequency stimulation (50 Hz, 20 stimuli) was utilized to deplete the readily releasable vesicle pool, subsequently, a test pulse at various intervals (0.1 s, 0.5 s, 1 s, 2 s, 5 s, 10 s) was used to examine the vesicles replenishment from depletion (Fig. 4E). As shown in Fig. 4F, SR58611A (15 µM) did not influence the time constant (Fig. 4F, control: 2.2 ± 0.2 s, SR58611A: 2.3 ± 0.1 s, n = 7 cells from six rats, paired t-test, P > 0.05 vs. controls), suggesting SR58611A (15 µM) has no effect on the rate of recovery from vesicle depletion.

## Discussion

**Figure 5:**
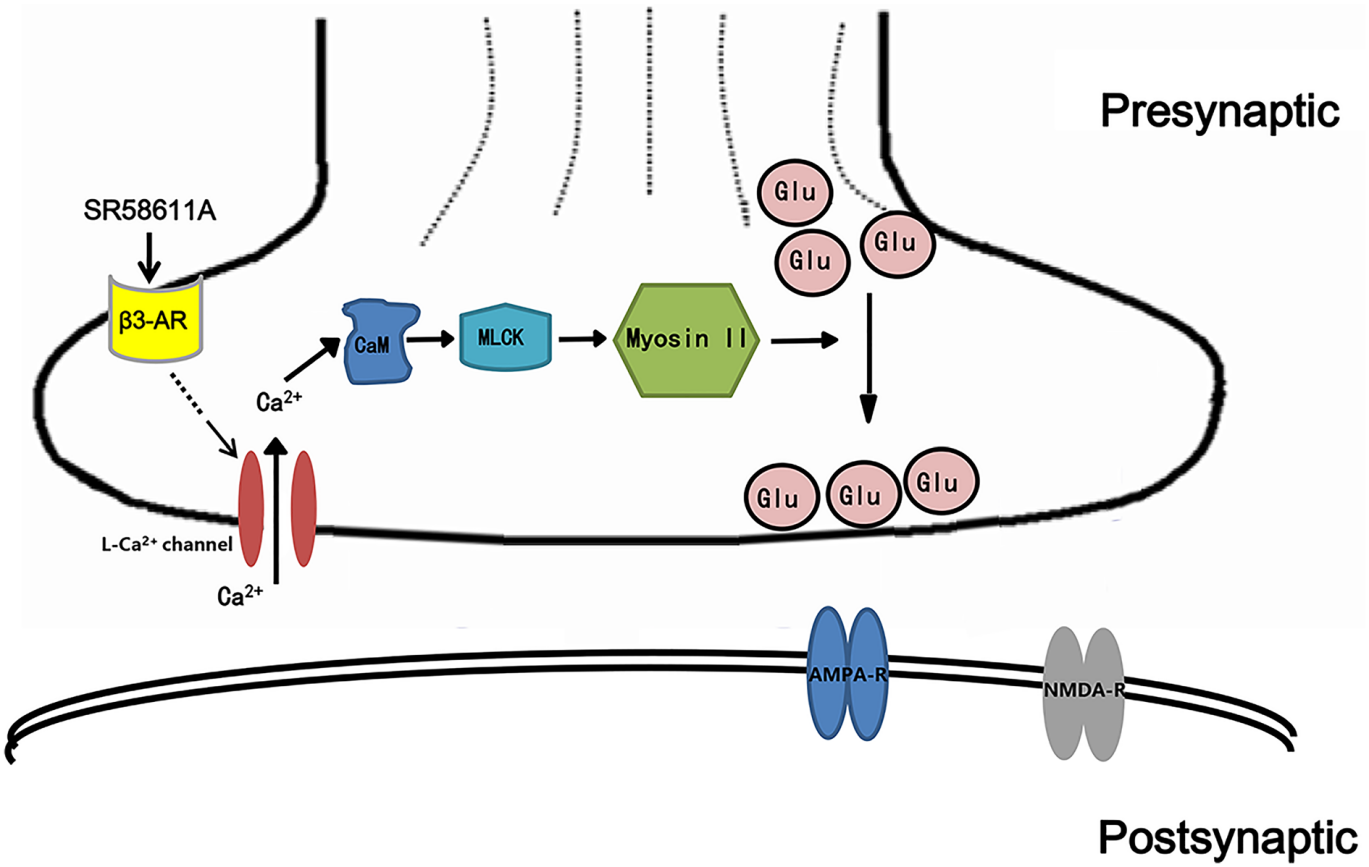
A working hypothesis of the mechanism based on the available evidence. Activation of pre-synaptic β3-AR results in increases in intracellular Ca^2+^ concentration via activation of L-type Ca^2+^ channel, leading to activation of MLCK. MLCK further phosphorylates and changes the conformation of myosin II, a motor protein, to facilitate the delivery of vesicles from the reserve pool to the readily releasable pool to increase glutamate release.

Our current result demonstrates that SR58611A (15 µM) increases AMPAR-EPSCs in the PFC via activating β3-AR, and L-type Ca^2+^ channel plays an important role in SR58611A-enhanced glutamate release. According to our current data, we speculate that activation of β3-AR results in increases in intracellular Ca^2+^ concentration via activation of L-type Ca^2+^ channel, leading to activation of MLCK. MLCK further phosphorylates and changes the conformation of myosin II, a motor protein, to facilitate the delivery of vesicles from the reserve pool to the readily releasable pool to increase glutamate release. A working hypothesis of the mechanism based on the available evidence has been proposed (Fig. 5).

Our result demonstrates that SR58611A (15 µM) enhances glutamatergic transmission via increasing pre-synaptic glutamate release without regulating post-synaptic AMPA receptor function because SR58611A (15 µM) only increases the mEPSC frequency without influencing the mEPSC amplitude. This is ensured further by the following evidence. First, SR58611A (15 µM) decreases PPR of AMPAR-EPSCs because increases in pre-synaptic vesicle release usually relates to reduction of PPR. Second, SR58611A (15 µM) has no effects on glutamate currents from the cell body of recorded pyramidal neurons indicating SR58611A (15 µM) does not directly regulate post-synaptic AMPA receptor. Third, SR58611A-mediated enhancement of AMPAR-EPSCs is still observed when a general G-protein inactivator GDP-β-S (1 mM) is contained in the recording pipettes to inhibit possible post-synaptic β3-AR function. According to our electrophysiological results, we speculate that SR58611A (15 µM) facilitates glutamate release via acting at β3-AR located on pre-synaptic glutamatergic terminals.

Whereas SR58611A (15 µM) facilitates glutamate release onto pyramidal neurons, the potential cellular and molecular mechanisms is unclear. Our current results demonstrate that extracellular Ca^2+^ influx through L-type Ca^2+^ channel contributes to SR58611A-induced increase in glutamate release. Consistent with our result, activation of β3-AR has been reported to stimulate L-type Ca^2+^ channel in rat vascular myocytes^19^. However, the precise mechanism underlying the activation of L-type Ca^2+^ channel by SR58611A is unknown. We consider two potential mechanisms to explain how SR58611A interacts with L-type Ca^2+^ channel. First, β3-AR activation directly interacts with L-type Ca^2+^ channel to open leading to influx of Ca^2+^. Second, β3-AR activation causes persistent pre-synaptic terminals depolarization, subsequently activates L-type Ca^2+^ channel resulting in potentiation of Ca^2+^ influx.

Our data indicates that SR58611A (15 µM) facilicates the number of releasable glutamatergic vesicles pool, and SR58611A-enhanced releasable glutamatergic vesicles number is blocked when calmodulin, MLCK or myosin II function is inhibited, respectively. Thus, it is reasonable to suppose that the Ca^2+^/calmodulin/MLCK/myosin II pathway is involved in the delivery of glutamatergic vesicle from the reserve pool to the readily releasable pool induced by β3-AR. Consistent with our hypothesis, it has been revealed that elevation of pre-synaptic calmodulin function enhances the number of readily releasable synaptic vesicle^27^. Calmidazolium, which is a calmodulin antagonist, abolishes nicotine-induced enhancement of the number of the releasable synaptic vesicle^28^. Moreover, there is convincing evidence that MLCK and myosin II are necessary for the synaptic vesicle mobilization at a variety of synapses in multiple brain regions^20, 22, 27^.

Studies from Mochida et al. and Ryan show that MLCK and myosin II are required for neurotransmission^20, 21^, whereas our current result shows that a similar treatment only blocks enhancement by SR58611A. The inconsistency may be due to different experimental materials. The electrophysiological data that we collected comes from neurons in brain acute slices of rat PFC, whereas the experimental materials of studies from Mochida et al. and Ryan are cultured neurons from superior cervical ganglion or hippocampus^20, 21^, which may result in distinct properties of functional MLCK and myosin II expression in neurons in culture and in acute slices.

Whereas our result shows that MLCK and myosin II are downstream target of β3-AR, Tamburella et al. reports that activation of β3-AR increase the hippocampal brain-derived neurotrophic factor (BDNF) expression^6^. Given that application of BDNF has profound effects on glutamatergic transmission^29^, it is possible that BDNF is involved in SR58611A-induced enhancement of glutamatergic transmission. Whereas our current data indicates that SR58611A regulates glutamatergic signaling, a study from Consoli et al. (2007) has revealed that 5-HT antagonist methysergide (2 mg/kg) preventes the antidepressant- and anxiolytic-like activity of SR58611A indicating that 5-HT transmission is strictly involved in its action^30^. Further experiments will be designed to test how SR58611A modulates 5-HT transmission in the PFC.

Because SR58611A exhibits antidepressant- and anxiolytic-like effects, and enhances memory formation^5, 31^. Furthermore, SR58611A-induced enhancement of glutamate release resembles long-term potentiation (LTP). Therefore, it is reasonable to speculate that SR58611A-mediated augmentation of glutamate release may serve as a cellular and molecular mechanism to explain its roles in facilitating behavioral changes. Given that β3-AR activation enhances memory formation, it is not irrational to conjecture that activation of the β3-AR in the soma or dendrites of PFC neurons increases neuronal excitability which is then propagated to the glutamatergic terminal, Ca^2+^ influx via L-type Ca^2+^ channel in response to SR58611A-induced depolarization would activate calmodulin and MLCK to increase the readily releasable pool size resulting in increases in glutamate release. Facilitation of glutamatergic transmission in the PFC circuitry likely augments behaviors and memory formation. Our results in this study, therefore, at least provide a potential cellular and molecular mechanism whereby SR58611A improves memory formation and possesses antidepressant- and anxiolytic-like effects.

## Materials and methods

### Animals

The experiment was approved by Ethical Committee on Animal Experiment Committee of Nanchang University (KY-20200601). Male or female Spraque-Dawley rats (14–23 days after postnatal, P14–P23) were housed singly in a 12 h light/dark cycle in a temperature- and humidity-controlled environment with food and water freely available. All experiments and methods were performed in accordance with the Animal Research: Reporting of In Vitro Experiments (ARRIVE) guidelines. We declared that all methods were carried out in accordance with the relevant guidelines and regulations.

### Slices preparation

Animals were anesthetized with sodium pentobarbital (40 mg/kg, i.p.). PFC slices were prepared according to the procedures we described previously^17^. Briefly, the brain was removed rapidly from the skull and brains were dissected out in artificial cerebrospinal fluid (ACSF) containing 124 mM NaCl, 25 mM NaHCO_3_, 10 mM glucose, 3 mM KCl, 2 mM CaCl_2_, 1.5 mM MgSO_4_, 0.4 mM ascorbic acid, and saturated with 95% O_2_ and 5% CO_2_ at ~ 0 °C. With the pH set to 7.4. Five to six horizontal 350 μM slices containing PFC were cut on a vibratome (VT-1200, Leica, Wetzlar, Germany) and transferred to ACSF at room temperature until use. Slices were incubated for at least 40 min before recording.

### Whole-cell patch clamp recordings

A single slice was transferred to the recording chamber and fixed at the bottom of chamber using a nylon grid glued to the platinum frame. Recordings were performed utilizing patch pipettes (3–7 MΩ) containing150 mM K^+^ gluconate, 8 mM NaCl, 10 mM HEPES, 0.4 mM EGTA, 2 mM ATP. Mg, 0.1 mM GTP.Na^+^_3_, and 10 mM Na2phosphocreatine with pH adjusted to 7.4 by KOH, and with an osmolarity of 290–320 mOsm.

For recording of mEPSCs, theGABA_A_ receptor Picrotoxin (PTX, 100 µM) was included in the extracellular solution to block GABAergic transmission onto the recorded cell, and Tetrodotoxin (TTX, 1 µM) was bath applied to abolish spontaneous action potentials. To record AMPAR-EPSCs, a bipolar stimulation electrode was positioned approximately 150–200 µm from the apical dendrites of recorded cell. Stimulations were delivered at 0.033 Hz using Master-8 (A.M.P. Instruments Ltd, Jerusalem, Israel) in the extracellular presence of PTX (100 µM) and NMDA receptor antagonist AP-5 (50 µM). To record glutamate-induced currents, glass pipettes (the same as recording pipettes) containing glutamate (100 µM) was placed near the cell body of recorded cell in the extracellular presence of PTX (100 µM), AP-5 (50 µM) and TTX (1 µM). Cyclothiazide (CTZ, 10 µM) was additionally included in the bath to block AMPA receptor desensitization. Glutamate was applied through pressure ejection utilizing a pneumatic picopump.

### Drug application

SR58611A, SR59230A, betaxolol, ICI118551, anisomycin, BAPTA-AM, L-glutamic acid potassium salt, thapsigargin, nimodipine, verapamil, W7, wortmannin, ML-9, PTX, ATP. Mg, GTP.Na^+^_3_, HEPES and AP-5 were purchased from sigma chemical company (Sigma, St Louis, MO, USA). CTZ was obtained from tocris cookson Ltd (Ellisville, MO, USA). TTX was obtained from the research institute of aquatic products, Hebei province, China.

### Data analysis

Baseline recorded 5 min prior to application of SR58611A was selective as control condition. Effect of SR58611A was assessed from 5 to 10th min after the start of SR58611A application. For AMPAR-EPSCs and glutamate-induced currents, the amplitudes before and during SR58611A application were compared statistically using a 2-tailed paired student’s t-test. An analysis of variance followed by post-hoc tests was used to multiple comparisons between groups.

Concentration–response curve of SR58611A was fitted by Hill equation: y = I_max_ × {1/[1 + (EC_50_/[SR58611A concentration])^n^]}, where I_max_ is the maximal facilitation effect, EC_50_ is the concentration of SR58611A producing a half maximal enhancement concentration, and n is the hill coefficient. For mEPSCs, successive events recorded were detected automatically with a threshold-crossing algorithm, and the frequency and amplitude of the events were calculated using the mini analysis program. The PPR was assessed as the mean EPSC2/mean EPSC1, where EPSC1 was the first evoked current amplitude and EPSC2 was the second synaptic current, measured after subtraction of the remaining EPSC1 ‘tail’ current^32^. All data are expressed as means ± standard error of the mean (SEM) in the text and figures. Statistical significance was assessed at P < 0.05. Asterisks in the figures indicate positive significance levels and n refers to the number of neurons examined.

### Ethics approval

The experiment was approved by Ethical Committee on Animal Experiment Committee of Nanchang University.
